# 3D genome topology distinguishes molecular subgroups of medulloblastoma

**DOI:** 10.1016/j.ajhg.2024.10.003

**Published:** 2024-10-30

**Authors:** John J.Y. Lee, Michael J. Johnston, Hamza Farooq, Huey-Miin Chen, Subhi Talal Younes, Raul Suarez, Melissa Zwaig, Nikoleta Juretic, William A. Weiss, Jiannis Ragoussis, Nada Jabado, Michael D. Taylor, Marco Gallo

**Affiliations:** 1 The Arthur and Sonia Labatt Brain Tumor Research Center, The Hospital for Sick Children, Toronto, ON M5G 0A4, Canada; 2 Developmental & Stem Cell Biology Program, The Hospital for Sick Children, Toronto, ON M5G 0A4, Canada; 3 Department of Laboratory Medicine and Pathobiology, University of Toronto, Toronto, ON M5G 1L7, Canada; 4 Cumming School of Medicine, University of Calgary, Calgary, AB T2N 4N1, Canada; 5 Department of Pediatrics, Baylor College of Medicine, Houston, TX 77030, USA; 6 Cancer and Hematology Center, Texas Children’s Hospital, Houston, TX 77030, USA; 7 Department of Human Genetics, McGill University, Montreal, QC H2A 1B1, Canada; 8 Department of Pediatrics, McGill University, The Research Institute of the McGill University Health Centre, Montreal, QC H4A 3J1, Canada; 9 Division of Experimental Medicine, Department of Medicine, McGill University, Montreal, QC H3A 3J1, Canada; 10 Department of Neurology, University of California, San Francisco, San Francisco, CA 94143, USA; 11 Department of Pediatrics, University of California, San Francisco, San Francisco, CA 94143, USA; 12 Department of Neurosurgery, University of California, San Francisco, San Francisco, CA 94143, USA; 13 Brain Tumor Research Center, and Helen Diller Family Comprehensive Cancer Center, University of California, San Francisco, San Francisco, CA 94143, USA; 14 McGill Genome Centre, Montreal, QC H3A 0G1, Canada; 15 Dan L Duncan Comprehensive Cancer Center, Baylor College of Medicine, Houston, TX 77030, USA

**Keywords:** Hi-C, 3D genome, transcriptome, medulloblastoma, cancer, CNS tumor

## Abstract

Four main medulloblastoma (MB) molecular subtypes have been identified based on transcriptional, DNA methylation, and genetic profiles. However, it is currently not known whether 3D genome architecture differs between MB subtypes. To address this question, we performed *in situ* Hi-C to reconstruct the 3D genome architecture of MB subtypes. In total, we generated Hi-C and matching transcriptome data for 28 surgical specimens and Hi-C data for one patient-derived xenograft. The average resolution of the Hi-C maps was 6,833 bp. Using these data, we found that insulation scores of topologically associating domains (TADs) were effective at distinguishing MB molecular subgroups. TAD insulation score differences between subtypes were globally not associated with differential gene expression, although we identified few exceptions near genes expressed in the lineages of origin of specific MB subtypes. Our study therefore supports the notion that TAD insulation scores can distinguish MB subtypes independently of their transcriptional differences.

## Graphical abstract

**Figure:**
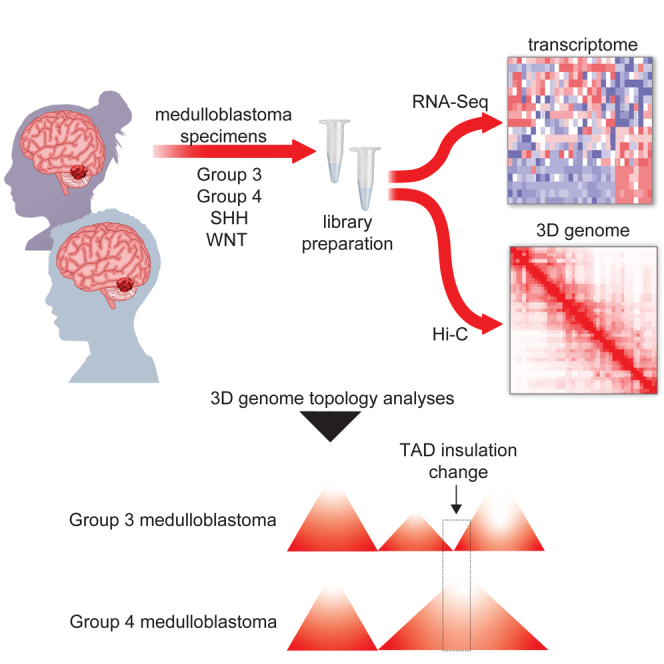

## Teaser

Hi-C profiling of brain tumor samples identified topologically associating domain boundary differences between molecularly defined medulloblastoma subgroups. These results indicate that 3D genome topology represents an additional layer of heterogeneity between medulloblastoma subgroups.

## Introduction

Medulloblastoma (MB) comprises a heterogeneous group of tumors of the cerebellum that can be classified into four main molecular subgroups based on their characteristic transcriptional and mutational profiles: WNT, SHH, group 3 (G3), and group 4 (G4).^1^ WNT and SHH tumors are characterized by mutations that lead to activation of the Wingless and Sonic Hedgehog signaling pathways, respectively. The underlying genetics of G3 and G4 tumors is less well defined, although ∼17% of G3 tumors have *MYC* (MIM: 190080) amplifications and ∼6% of G4 tumors have amplifications of *MYCN* (MIM: 164840) (reviewed in Roussel and Robinson^2^). Additionally, about 40% of G4 tumors harbor somatic mutations that affect the function of the core binding factor alpha complex and have high frequency of isochromosome 17q.^3^^,^^4^^,^^5^

G3 and G4 tumors have transcriptional profiles that set them clearly apart from the other subgroups. However, single-cell RNA-sequencing (scRNA-seq) studies have also indicated that G3 and G4 tumors exist along a transcriptional continuum with some intermediate transcription profiles shared by both subgroups.^6^ Therefore, transcriptomic approaches are not sufficient to unambiguously distinguish these two subgroups. On the other hand, DNA methylation profiling has been successful at distinguishing all MB subgroups, including G3 and G4,^7^ highlighting the utility of this method for precise clinical diagnoses of brain tumors. This latter finding also indicates that epigenetic landscapes might better distinguish these MB subtypes than transcriptional or genetic profiles.

Molecular subgrouping of brain tumors has been an important tool to discriminate between entities that were previously considered pathologically similar but that in fact have different clinical profiles, including responses to therapy.^8^^,^^9^ Among MB subgroups, WNT tumors have extremely good prognoses, with the vast majority of individuals essentially cured of the disease. At the other end of the spectrum are G3 and SHH tumors, which have the worst prognoses among MBs.^10^ G4 tumors have intermediate prognoses. The classification of MB into molecular subgroups has represented a significant improvement over previous categorizations based on cell morphology and other pathology features. The widespread acceptance of this classification system has led to their inclusion in the World Health Organization Neuro-oncology classification.^11^

MB subgroups originate from distinct cell lineages of the developing hindbrain.^3^^,^^12^^,^^13^ SHH MBs likely originate from granule cell precursor lineages, whereas G3 and G4 tumors originate from progenitor cells that emanate from the ventral zone and subventricular zone (SVZ) of the rhombic lip. The origin of both G3 and G4 MBs from the rhombic lip at least partially explains that G3 and G4 tumors share some molecular profiles. G3 tumors are enriched for a photoreceptor gene signature that distinguishes them from a unipolar brush cell (UBC) signature that is more enriched in G4 tumors.^14^ Of note, UBC progenitors are particularly numerous in humans compared to other species, and they originate from a rhombic lip structure that is unique to human neurodevelopment.^3^ These recent findings may explain why previous attempts at modeling G3 and G4 MBs in murine mouse models have had limited success at recapitulating salient characteristics of these diseases.

Although several studies have investigated the genomic and transcriptomic landscape of MB subgroups, there remains a lack of knowledge regarding their epigenomes and 3D genomes. Recent work has described the potential for structural variants (SVs) to rewire the epigenome of MBs, especially through hijacking of cis regulatory elements (CREs).^5^ However, such epigenetic rewiring was inferred by overlaying SVs to 3D genome information generated from unrelated cell models. Systematic studies of 3D genome configurations across MB subgroups using clinical samples have not yet been described.

Here, we have used *in situ* Hi-C^15^ to map the 3D genome architecture of clinical and preclinical specimens across MB subgroups. The Hi-C contact maps we generated for our MB samples achieved high resolution and enabled the identification of all major features of the 3D genome.^16^ At the highest resolution, we were able to identify DNA loops, which have been reported to link CREs and their target genes and promoter-promoter interactions. Loops are often—but not necessarily—anchored by CTCF and originate from the motor function of the cohesin complex, which pulls DNA through its ring structure until a barrier to movement is encountered, often CTCF itself.^17^^,^^18^ The mechanism mediated by cohesin and CTCF has been defined as loop extrusion. At the larger scale of 3D genome structures are topologically associating domains (TADs), which represent contiguous genomic regions that tend to interact with each other more often than with surrounding regions.^16^ The majority of TADs are delimited by CTCF proteins that bind their cognate DNA motifs in convergent orientation.^16^

Here, we present Hi-C contact maps of MB specimens spanning all four main molecular subgroups. Using these datasets, we highlight fundamental 3D genome differences between MB subgroups, including G3 and G4 tumors.

## Material and methods

### Human samples

Primary tumors used in the study were collected and processed after receiving written informed consent, including consent to publish the generated data, as per guidelines from Research Ethics Board from the following institutes: Hospital for Sick Children (Toronto, Canada), McGill University (Montreal, Canada), and University of California, San Francisco (San Francisco, USA).

### Hi-C library preparation

Fresh tissue samples were obtained from The Hospital for Sick Children (Toronto, ON). *In situ* Hi-C libraries were generated as we described previously,^19^ using approximately 2.5 million dissociated cells as input. All Hi-C libraries were sequenced at 150 bp PE with a Hi-Seq X instrument (Illumina) at McGill Genome Centre (Montreal, QC).

### Hi-C contact maps

Juicer (v1.6, CPU)^20^^,^^21^ was used to process Hi-C library fastqs to “.hic” format contact maps. Dependencies of the Juicer pipeline included bwa (v0.7.17)^22^ and Java (openjdk = 8.0). Reads were aligned using hg38 coordinates (GCA_000001405.15_GRCh38_no_alt_plus_hs38d1_analysis_set). All samples were confirmed to have resolution <15 kb (as determined by the Juicer script “calculate_map_resolution.sh”) and >10% of alignable read pairs resulting in long-range contacts (as determined from Juicer’s inter_30.txt QC file) prior to proceeding with downstream analysis. For in-depth downstream methods, please see Johnston et al.^19^ Loop anchors were analyzed with the R package ChIPSeeker.^23^^,^^24^ Coordinates for loop anchors were annotated using the UCSC.hg38.knownGene annotations. Annotation of contact domains, boundaries, loops, and compartment was done as described in Johnston et al.^19^ and as summarized below.

#### Contact domains

To annotate contact domains, hic files were processed using Juicer Tools (v1.19.02, https://github.com/aidenlab/JuicerTools) Arrowhead with parameters “--ignore-sparsity -k SCALE” for the following data resolutions (kb): 10, 25, 50, and 100. To compare Arrowhead block scores between samples, we first defined the union of all domains called across all samples. Arrowhead was subsequently rerun with the parameters “feature_list” and “control_list” set to this domain union to calculate block scores at all positions of interest for each sample.

#### Boundaries

To assess boundary scores at the edges of contact domains, RobusTAD (v1.0) was run with parameters “--norm=norm” on 50-kb Hi-C contact matrices generated by Juicer Tools dump with parameters “-d observed SCALE BP 50000.”

#### Loops

To annotate chromatin loops, hic files were processed using Juicer Tools (v1.19.02) HiCCUPS with parameters “--cpu -m 4096 --ignore-sparsity -k SCALE” for resolutions (kb): 10 and 25. To compare HiCCUPS loop scores between samples, we first defined the union of all loops called across all samples. HiCCUPS was subsequently rerun with the parameter “specified_loop_list” set to this loop union to calculate loop scores at all positions of interest for each sample.

#### Compartments

To annotate chromatin compartments, hic files were processed using Juicer Tools (v1.19.02) Pearsons with parameters “SCALE BP 50000 -p” to return the correlation matrix for each chromosome. Eigenvectors for principal components 1–3 of the Pearson correlation matrix were calculated in R (v4.0.3; https://www.R-project.org/) and separated into their positive and negative values, and each was compared to brain H3K27ac ChIP signal (http://www.roadmapepigenomics.org/data) using “bedtools jaccard” (v2.26.0). The eigenvector with the highest jaccard similarity was selected to represent genome compartmentalization. If the highest jaccard similarity corresponded to the negative values of the eigenvector, then the eigenvector was inverted such that positive values correspond to type A compartmentalization.

### SNF clustering

Similarity network fusion (SNF)^25^ clustering was performed with the package that can be found at https://github.com/maxconway/SNFtool. For boundaries (RobusTAD) and compartments (eigenvector), feature scores were first filtered to include only positions where <5% of samples resulted in undefined calls and positions that were among the top 40% of variance across samples. Inter-sample distances were then calculated using the squared Euclidean distance as recommended by the SNF manual for continuous values.

For domain calls (Arrowhead) and loop calls (HiCCUPS), the numerical values of the feature scores were found to be noisier than the binary presence or absence of the feature call, therefore the Jaccard distance between called features was used.

Next, the affinity matrix for each feature was calculated with K = 20 and sigma = 0.3. The overall fused matrix between these affinity matrices was calculated with K = 20 and T = 16. The distance between samples was taken as (0.5 – similarity). Finally, these distances were plotted using UMAP with n_components = 2. Concordance of individual features with the final fused matrix were generated using concordanceNetworkNMI with C = 2. Distance between annotated features was taken as (1 – concordance).

### Transcriptome analysis

#### Library preparation

RNA-seq libraries were generated and sequenced at the Ontario Institute for Cancer Research using Kapa RNA HyperPrep kit. Three RNA-seq libraries were pooled per lane of HiSeq 2500 High-output PE126.

#### Differential expression

RNA-seq reads were aligned to hg38 (GCA_000001405.15_GRCh38_no_alt_plus_hs38d1_analysis_set) using HISAT2 (v2.1.0)^26^ with parameters “--rna-strandness RF --downstream-transcriptome-assembly.” SAM output was sorted, converted to BAM, and indexed using samtools (v1.9).^27^ A counts table was prepared using the count function of HTSeq (v0.13)^28^ and differential expression testing was performed using limma with Voom transformation (v3.44).^29^ Overlap between genomic features was performed using “BEDTools intersect.”^30^

### Comparison to single-cell transcriptomics of MB

Data were accessed from Riemondy et al.^31^ via https://www.pneuroonccellatlas.org/and visualized using the UCSC Single Cell Browser.^32^

### General statistical analysis and plotting

Data visualization was performed primarily in R (v4.0.3). Libraries used include broom (v0.7.5), ggpubr (v0.4.0), ggrepel (v0.9.1), RColorBrewer (v1.1.2), and tidyverse (v1.3.0). Non-negative matrix factorization (NMF) dimension reduction was run with library NMF (v0.21.0) in R 3.6.3. RobusTAD was run with library optparse (v1.6.6) in R 3.6.3. Visualization of Hi-C contacts and annotated features was performed using Juicebox (v1.11.08), HiCExplorer (v3.7.2),^33^ pyGenomeTracks (v3.6),^34^ or IGV (v2.12.3).^35^ Copy number and transcriptional analyses of *PRDM6* were generated with data from previously published SNP6 arrays^36^ and previously published gene expression array data^37^ processed as described in Hendrikse et al.^3^ *p* value was calculated by using two tailed Mann Whitney U test. The *PRDM6* expression plot was generated using just G4 MB samples with expression array and SNP6 array data available.

### Association between differential boundaries and differential expression

“BEDTools reldist” (v2.26.0)^30^ was used to assess the relative distance between significantly differentially expressed genes and significantly differential boundary scores. “BEDTools closest” was used to identify both the closest upstream and downstream boundary to each gene. “BEDTools window” was used to identify all boundaries within 1 Mb of each gene, then the most significantly differential boundary within this range was assigned to each gene.

### Linked-read whole-genome sequencing (10× Genomics)

Linked-read whole-genome sequencing data for MB samples was reported in our previous publication,^38^ and the methods are summarized here. High molecular weight DNA was extracted from tumors using phenol chloroform extractions. Molecule length was assessed by Femto Pulse (Genomic DNA 165 kb Kit, 3 h run, Agilent Technologies, Santa Clara, California, United States, cat# FP-1002-0275), and samples were quantified in triplicate using the Qubit dsDNA HS Assay Kit (Thermo Fisher Scientific, cat# Q32854). Library preparation was done following the Chromium Genome Reagent Kits v2 User Guide (10× Genomics, Pleasanton, California, United States) and sequenced using 150 PE Illumina reads. Data were aligned to reference genome b37 using 10× Genomics’ pipeline (LongRanger; https://github.com/10XGenomics/longranger). Samples were sequenced to a mean depth of 40× with an average of 942 million reads. Mean molecule length for the samples was 21.3 kb and resulted in the detection of an average of 362 large SV calls and 4,643 short deletion calls.

## Results

### 3D genome reconstruction and transcriptional profiles of MB samples

**Figure 1:**
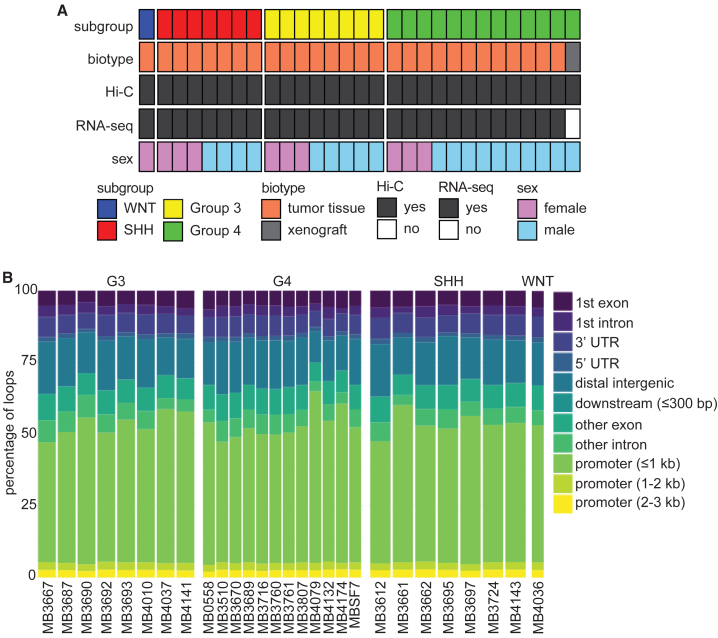
3D genome and transcriptome analyses of MB subgroups (A) Summary of the MB samples used for the present study. Molecular subgroup, biotype, and biological sex are indicated for each specimen profiled by Hi-C. The successful generation of matched RNA-seq data is also indicated for each specimen. (B) Bar plot showing the percentage of loop anchors that fall within different types of genomic annotations.

We aimed to characterize the 3D genomes of different MB molecular subgroups and to investigate their relationships to subgroup-specific transcriptional profiles. We generated *in situ* Hi-C contact maps for 29 samples, including 28 surgical specimens and one patient-derived xenograft (Figure 1A). In addition, we generated RNA-seq data for the 28 surgical samples to enable downstream correlation of 3D genome architecture and transcriptional profiles. The MB specimens profiled include 1 WNT, 7 SHH, 8 G3, and 13 G4 samples (Figure 1A; Table S1). Two G4 samples represent the diagnostic (MB3670) and recurrent (MB4079) tumors from the same individual. Given that only one WNT MB sample was available, our analysis focuses primarily on the contrasts between G3, G4, and SHH MBs. Ten samples were from female individuals, and 19 were from males, reflecting the prevalence of G3 and G4 samples in our cohort, which are known to have a 2:1 male:female ratio. The sex ratio among SHH samples was balanced, as expected for this subtype.

**Figure 2:**
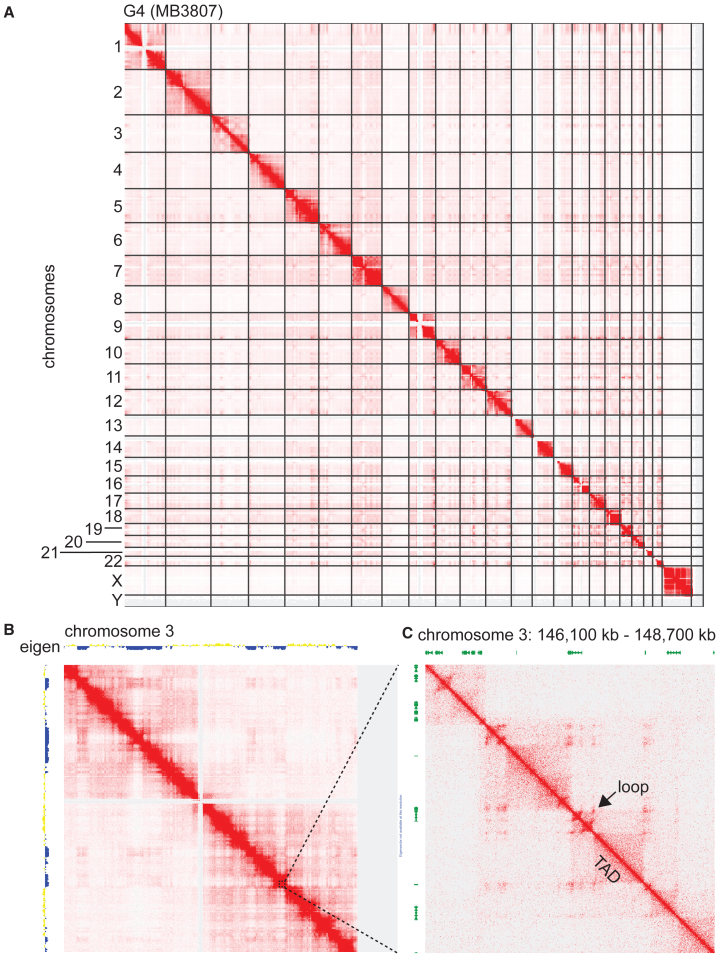
Example of MB Hi-C data (A) Hi-C contact map for all chromosomes for sample MB3807, a G4 MB. Data for all chromosomes are displayed to enable visual detection of putative intra- and inter-chromosomal physical interactions between genomic regions. (B) Hi-C contact map for chromosome 3 of MB3807. Eigenvalues are represented along both the x and y axes, where yellow (positive) values identify type A compartments, and blue (negative) values correspond to type B compartments. (C) Enlarged image from (B) illustrates the use of Hi-C data to visualize TAD and loop structures. This region corresponds to 2,600 kb of genome on chromosome 3. Annotated genes are displayed in green along both the x and y axes of the heatmap. Computationally inferred loops are clearly visible off the diagonal, as indicated by the arrow in one example. TADs are visible as “triangles” as indicated in one example.

The Hi-C libraries had an average resolution of 6,833 bp (high: 3,950; low: 12,250 bp; median: 6,550 bp; Table S2), which allows robust visualization and analyses of all major elements of 3D genome structure. Globally, loops were annotated using HiCCUPS and TADs were annotated using Arrowhead (Tables S3 and S4). About half of all loops detected in each sample were anchored in proximal promoter regions, irrespective of molecular subgroups (Figure 1B). The number of loops (Figures S1A and S1B) and TADs (Figures S1C and S1D) detected were approximately uniform across MB subgroups. On the other hand, we found that TADs were shorter in G3 than in G4 (Wilcoxon test *p* = 0.0031; Figure S1E) and SHH (*p* = 1.2 × 10^−6^; Figure S1E) tumors. Similarly, TADs in G4 were shorter than in SHH tumors (*p* = 0.0094; Figure S1E). These differences were small but statistically significant. TAD size distribution for the WNT subgroup is shown, but these were not compared to other subgroups because we included only one WNT sample in our cohort. Globally, genome topologies appear relatively stable across MB subgroups. As an illustrative example of global 3D genome data, we display Hi-C contact maps for all chromosomes generated with the G4 MB sample MB3807 (Figure 2A). Overlaying Hi-C maps for an individual chromosome (chromosome 3 in this example) with eigenvector values, it is possible to recognize compartments (Figure 2B). Positive eigen values coincide with type A compartments (generally open chromatin), and negative eigen values correspond to type B compartments (compact chromatin). When looking at smaller regions, Hi-C contact maps enable the visualization of individual TADs and loops (Figure 2C). The MB Hi-C datasets we generated are therefore suitable for exploration of all levels of 3D genome architecture.

### TAD insulation scores distinguish MB subgroups

**Figure 3:**
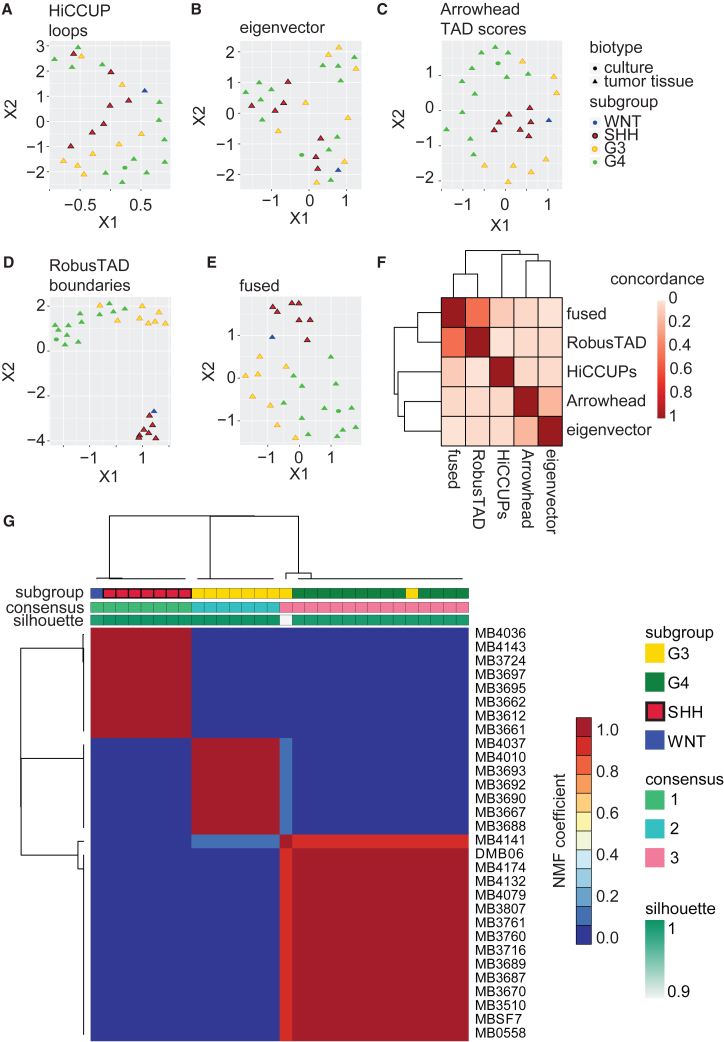
TAD insulation scores distinguish MB molecular subgroups (A) UMAP plot of MB samples based on loops called with HiCCUP. (B) UMAP plot generated with eigenvector values, which provide information on compartments. (C) UMAP plot generated with TAD scores called with Arrowhead. (D) UMAP plot generated with boundary scores calculated with RobusTAD. (E) UMAP plot based on fused distance metrics. (F) Concordance between metrics derived from Hi-C data described in this study. (G) Non-negative matrix factorization (NMF) analysis of TAD insulation scores for all MB samples, with k = 3. MB subgroups (G3, G4, SHH, and WNT) are indicated at the top of the image, highlighting the ability of TAD insulation scores to discriminate between subgroups. Consensus clusters are shown in green, blue, and pink. Silhouette scores provide the confidence level for the clustering calls for each sample.

We asked whether 3D genome information can differentiate between MB subtypes. We first tested loops, which failed to group subtypes, as shown in UMAP (Uniform Manifold Approximation and Projection) plots (Figure 3A). We then tested eigenvector values, which represent open type A compartments (positive values) or closed type B compartments (negative values). Compartmentalization also failed to properly group tumor subtypes (Figure 3B), consistent with the notion that genome compartmentalization is relatively stable across MB subtypes. On the other hand, Arrowhead TAD scores produced distinct clusters of SHH, G3, and G4 samples in UMAP plots (Figure 3C). These data suggested that MB subgroups may be distinguished by their TAD boundaries. We therefore computed TAD boundary scores genome wide for all samples in our cohort using RobusTAD^39^ (Table S5). UMAP plots show that TAD boundaries can cluster MB samples based on their molecular subtypes (Figure 3D). This clustering was more defined than when TAD scores were used. Finally, we wanted to see if integrating all three major 3D genome structures could achieve better stratification than looking at individual metrics. We applied SNF^25^ to integrate the signals from all measured 3D genome features (loops, eigenvector/compartments, TAD scores, and TAD boundaries) into a single, fused similarity matrix. UMAP plots using the fused distance metric achieved separation of samples based on their molecular subtype (Figure 3E). However, subgroup clustering using the fused distance was less pronounced than using the TAD boundary scores alone. The concordance between metrics was highest for TAD boundary scores and fused (Figure 3F). Overall, these results indicate that TAD boundary score is the 3D genome feature that is most predictive of MB molecular subtype, including the ability to distinguish between G3 and G4 tumors.

To further investigate the ability of TAD boundary scores to distinguish between tumor subtypes, we performed NMF analysis of TAD insulation scores for all MB samples. Choosing k = 3 resulted in clusters that largely recapitulated our previous observations and separated samples based on tumor subgroup (Figure 3G). Cluster 1 included all SHH samples and the single WNT sample in our collection. Cluster 2 included only G3 samples, and cluster 3 included all the G4 samples and two G3 samples. Our analyses therefore identified the TAD insulation score as a 3D genome metric that can distinguish between MB subgroups, including G3 and G4 MBs.

### Boundary strength differences between MB subgroups

We next aimed to identify the TAD boundaries that contribute to the differences between molecular subgroups. Toward this aim, we performed pairwise analyses of differential boundaries between SHH, G3, and G4 subgroups.

**Figure 4:**
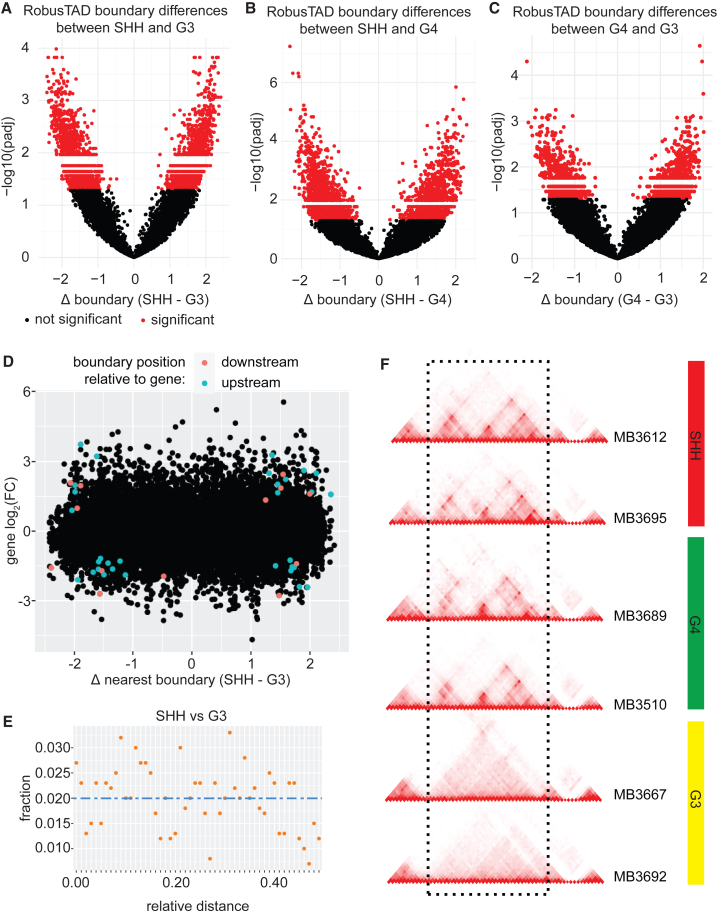
TAD boundary differences between MB subgroups (A) Volcano plot displaying differential TAD boundaries between SHH and G3 MBs. Negative log_10_ of the adjusted *p* value (padj) are displayed along the y axis. (B) Volcano plot displaying differential TAD boundaries between SHH and G4 medulloblastomas. (C) Volcano plot displaying differential TAD boundaries between G4 and G3 medulloblastomas. (D) Relationship between gene expression and differences in boundary strengths between SHH and G3 MB. Each dot represents a gene. Colored dots represent differentially expressed genes between SHH and G3. Pink dots represent genes that are differentially expressed and have a differential boundary downstream. Blue dots represent genes that are differentially expressed and have a differential boundary upstream. (E) Relationship between differentially expressed genes and their distance from differential TAD boundaries between SHH and G3 samples. Blue dashed line represents the expected frequency of observations based on 50 kb bins. Orange points represent the observed frequencies. (F) Example of differential boundaries between G3 and other MB subgroups. Two SHH samples, two G4 samples, and two G3 samples are displayed. The dashed rectangle highlights a genomic region with locally different topology in G3 tumors compared to SHH and G4. Specifically, the TAD structures observed in SHH and G4 MB are lost in G3 samples.

2,803 boundaries out of a total of 13,885 were significantly different between SHH and G3 subgroups (two-sided t test, padj <0.05; Figure 4A; Table S6), 2,650 boundaries were significantly different between SHH and G4 samples (Figure 4B; Table S7), and 1,486 boundaries were significantly different between G3 and G4 samples (Figure 4C; Table S8). Overall, 20.2%, 18.8%, and 10.7% of TAD boundaries were significantly different between SHH and G3, SHH and G4, and G3 and G4, respectively.

We wondered if boundaries with differential strengths between subgroups were associated with differential expression levels of neighboring genes. In pairwise comparisons of MB subgroups, only 44 genes immediately upstream or downstream of these boundaries were differentially expressed between SHH and G3 samples (Figure 4D; Table S9), indicating that differential boundaries were not strongly predictive of changes in expression. To better visualize any potential trends, we plotted expression levels of genes against the relative distance of that gene from the differential boundary using the reldist function available with BEDTools.^30^ To visualize the relationships between gene expression and boundaries, we plotted the data for each pairwise comparison between subgroups along a relative distance from the closest TAD boundary: SHH vs. G3 (Figure 4E), SHH vs. G4 (Figure S2A), and G4 vs. G3 (Figure S2B). The results show no clear trend for the expression of genes in proximity of differential boundaries between any MB subgroup combinations. These results suggest that, on a global scale, TAD boundary strength differences between MB molecular subgroups do not have unequivocal effects on expression levels of nearby genes.

As an illustration of different boundary structure between subgroups, we present a region on chromosome 8 with drastically different TAD structures in some G3 MB samples compared to other subgroups (Figure S2C). Some samples have a “fragmented” architecture, with three TADs clearly visible, and other samples have a “fused” architecture, where the three TADs are replaced by a single, larger TAD. To contextualize these results, we compared Hi-C data from G3 samples (*n* = 2), G4 (*n* = 2), and SHH (*n* = 2). Both G3 samples had a fused architecture, whereas G4 and SHH had fragmented TAD arrangements (Figure 4F). These 3D structural differences between G3 and other subgroups were not a consequence of Hi-C library quality, including sequencing depth, as evidenced by QC data (Table S2) and by the clear visualization of TAD structures outside this region at comparable levels in G3, other MB subgroups, and non-neoplastic brain tissue.

### Differences in TAD boundaries between G3 and G4 MBs

**Figure 5:**
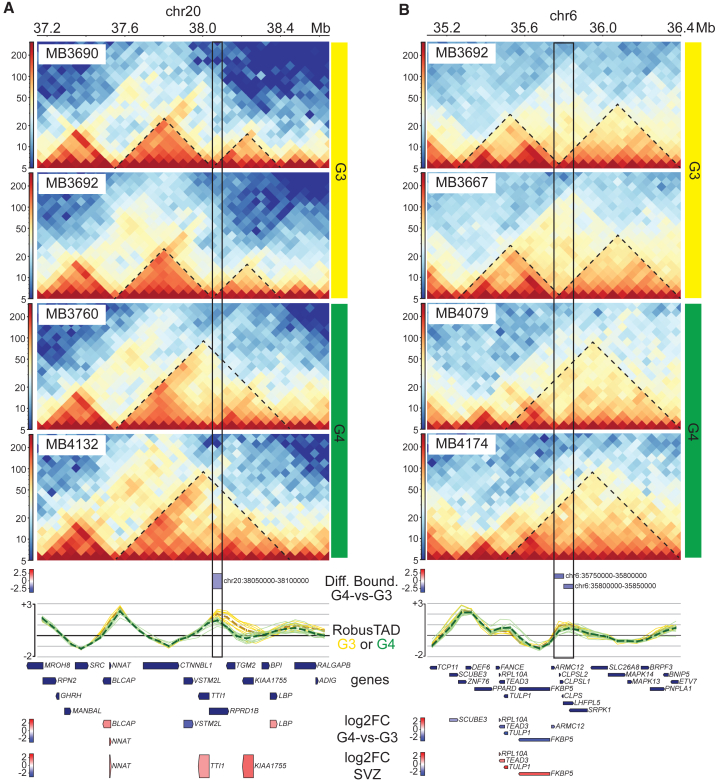
TAD boundary differences between G3 and G4 MB (A) Heatmap representing Hi-C contacts along a region of chromosome 20 (coordinates indicated at the top). Imputed boundary strengths along this region are shown as yellow and green lines for G3 and G4 tumors, respectively, below the heatmap. Gene expression levels are shown in the bottom diagram, including differential gene expression between G4 and G3 tumors. (B) Heatmap representing Hi-C contacts along a region of chromosome 6. Imputed boundary strengths along this region are shown as yellow and green lines for G3 and G4 tumors, respectively, below the heatmap. Gene expression levels are shown in the bottom diagram.

Given that TAD boundary scores differentiated between G3 and G4 tumors, we attempted to gain more insight into the underlying molecular underpinnings of this observation. Two recent papers described the origins of G3 and G4 tumors from lineages that emanate from the SVZ of the rhombic lip during early human embryonic development. Both tumor subgroups generally have gene expression profiles reminiscent of progenitor cells located in the rhombic lip SVZ.^3^^,^^14^ However, G3 tumors are enriched for a photoreceptor gene signature, whereas G4 tumors have transcriptional profiles reminiscent of UBCs.^14^ We therefore investigated the possibility that 3D genome architecture near genes associated with the lineage of origin of G3 and G4 tumors might distinguish these two MB subgroups. Accordingly, we intersected the genes identified in the rhombic lip SVZ photoreceptor cell signature^14^ with genomic loci exhibiting differential boundary strength between G3 and G4 samples based on our RobusTAD analyses. Here, we identified major 3D genome differences between G3 and G4 samples at key genes for their lineage of origin. In total, G3/G4 boundary differences were identified near one gene in the G3 lineage signature (*TULP1*, MIM: 602280; Table S10), and five genes that are part of the G4 lineage signature (*BARHL1*, MIM: 605211; *DDX3X*, MIM: 300160; *ZIC5*, MIM: 617896; *GJD2*, MIM: 607058; and *NNAT*, MIM: 603106; Table S11). For illustrative purposes, we generated contact maps at 50 kbp resolution for the *NNAT* genomic region on chromosome 20 for G3 and G4 tumors. Two representative contact maps for each tumor subtype are displayed in Figure 5A. In the BED track below the contact maps, we color-coded differential boundary calls based on the *Z* score difference between G4 and G3 so that shades of blue represent a boundary that is weaker in G4 than in G3 samples. Next, the line traces display the observed RobusTAD boundary scores for each of the G3 and G4 samples for each bin in each sample across the region. Boundary strengths for all G3 samples are shown as yellow lines, for all G4 samples as green lines, and dashed lines represent the average scores at each bin for each MB subtype. G3 tumors have a strong boundary at this site between two TADs. However, this boundary is weakened or lost in G4 tumors, resulting in fusion of the two TADs into a larger one. *NNAT* is transcribed at significantly higher levels in G4 compared to G3 tumors, and its expression is enriched in the SVZ in the Northcott data.^14^ We further corroborated these findings by interrogating scRNA-seq data published by the Vibhakar group (GEO: GSE156053 ^31^). These data include scRNA for one WNT, nine SHH, seven G3, and 11 G4 samples (Figure S3A). We confirmed that *NNAT* (Figure S3B) and its neighboring gene *BLCAP* (MIM: 613110) (Figure S3C) are strongly expressed in cells from G4 samples compared to other subgroups, although a few G3 cells also expressed these genes.

A second example is a differential boundary just upstream of *FKBP5* (MIM: 602623) and *TULP1*, two genes that are transcribed at higher levels in G3 tumors compared to G4 (Figure 5B). Transcription of both genes was significantly enriched in the SVZ based on RNA-seq data published by Northcott’s group^14^ and scRNA-seq from the Vibhakar group^31^ (Figures S3D and S3E). These data indicate that high transcriptional levels of *TULP1*, which is part of the photoreceptor gene signature, in G3 tumors is associated with increased strength of a boundary just upstream.

Both examples illustrate how 3D genome topology, specifically TAD organization, appears distinct between G3 and G4 tumors. The expansion of specific TADs in G4 samples could alter the regulatory landscapes accessible for regulation of downstream genes. These examples also imply deregulation of epigenome independently of SVs. Therefore, on a global scale, differential TAD boundaries are not associated with differential gene transcription levels. However, we identified a few differential boundaries between G3 and G4 samples that reconfigure 3D genome organization at genes that are differentially expressed between these two subgroups and that distinguish their cells of origin.

### Recurrent structural variants in G3 and G4 MBs

Hi-C data can be used to infer copy number and SVs.^40^ Deep deletions can be visualized from a Hi-C heatmap because of the lack of reads spanning a given region. SVs, like balanced translocations, for instance, appear as a characteristic “butterfly” shape on the Hi-C heatmap at the location off the diagonal that corresponds to the locations of the translocation partners, which can be located on the same chromosome or on different chromosomes. Hi-C data therefore can be used to infer both epigenomic context in 3D space and genetic landscapes of a given sample. Based on these premises, we aimed to interrogate our Hi-C data to identify SVs in G3, G4, and SHH MBs. We defined recurrent SVs and variants that affected overlapping genomic regions in at least two samples. Overall, we detected 107 putative recurrent SVs among our MB cohort (Figure S4A; Table S12). For example, we inferred a recurrent translocation involving chromosomes 12 and 16 in a G4 sample (MB3670; Figure S4B) and a G3 sample (MB3687; Figure S4C) with a conserved breakpoint about 50 Mb along chromosome 16. This translocation was validated with linked-read (10× Genomics) whole-genome sequencing in MB3670 (Figures S4D and S4E; estimated breakpoint at chr16: 50,092–50,094 kb).

**Figure 6:**
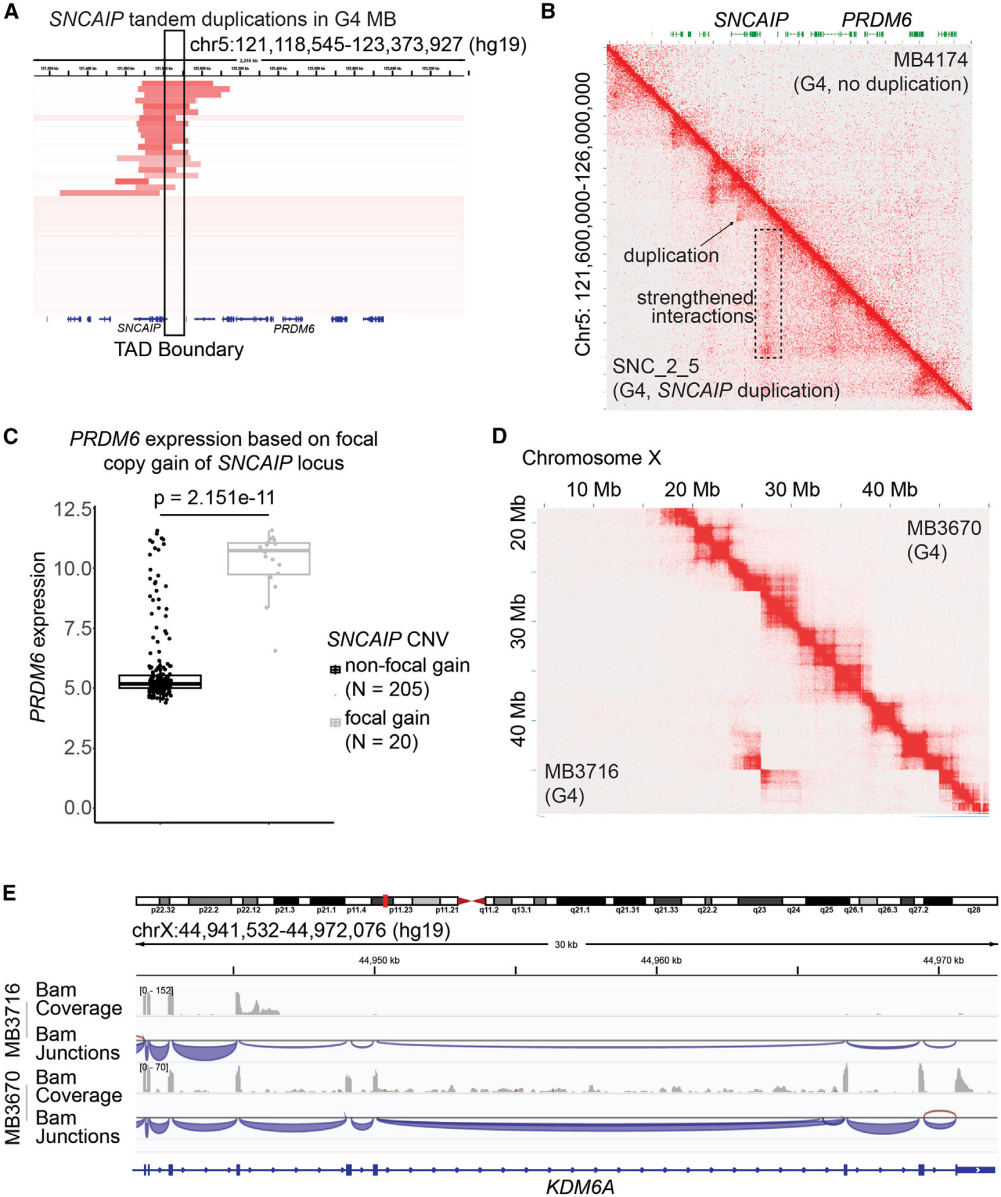
Relationships between SVs, 3D genome, and transcriptional profiles (A) Location of recurrent *SNCAIP* tandem duplications in the context of TAD boundaries in G4 MB. The coordinates of the region of chromosome 5 shown are indicated at the top. Copy number data for each MB sample are displayed along rows. MB samples with duplications are visible at the top of the diagram, with duplicated regions shown in red. The TAD boundary between the *SNCAIP* and *PRDM6* loci is highlighted with a black rectangle. (B) Observed minus expected (Obs-Exp) contact frequencies were determined by Hi-C data for a G4 MB with *SNCAIP* duplication (lower half) and for a G4 sample without the duplication (upper half). The genomic region represented is on chromosome 5 (coordinates shown along the y axis of the heatmap). (C) *PRDM6* transcriptional levels in samples with and without the *PRDM6* duplication. *p* value was calculated with the two-tailed Mann Whitney U test. (D) Inference of an SV affecting the *KDM6A* locus in a G4 MB (lower half of the Hi-C map). A G4 sample without SVs at this locus is displayed in the upper half of the heatmap. (E) RNA-seq data were used to determine the effects of the SV at the *KDM6A* locus on the transcriptional levels of this gene.

We found that the Hi-C contact map for one G4 sample was consistent with a previously described tandem duplication that included *SNCAIP* (MIM: 603779) and *PRDM6* (MIM: 616982)^36^ (estimated breakpoints at chr5: 122–123 kb). Given that previous papers inferred the effects of this duplication on 3D genome organization using Hi-C data from non-MB models, we thought that we now had a chance to contextualize the study of the effects of the duplication directly with our Hi-C data. This SV included duplication of an established super enhancer (SE) upstream of *SNCAIP* and recurrently affects a TAD boundary (Figure 6A). When we compared the Hi-C map for a sample with this putative translocation (lower half of Figure 6B) to another G4 sample without the *SNCAIP*-*PRDM6* tandem duplication (upper half of the heatmap in Figure 6B), we identified new patterns of physical interactions in the duplicated sample. This example demonstrates how genetic variants can result in reorganization of the 3D genome of cancer samples. The tandem duplication has significant effects on the transcriptional levels of *SNCAIP*, with duplication-positive samples having orders of magnitude higher expression of this gene compared to non-duplicated samples (Figure 6C). Overall, our Hi-C datasets provide insight into how genetic variation shapes 3D genome organization to achieve oncogenic transcription profiles.

About 4% of MB samples harbor truncating mutations, splice site mutations and missense mutations at the *KDM6A* locus.^41^ *KDM6A* is expressed at significantly lower levels in G3 and G4 than in SHH tumors (pairwise Welch t test *p* = 2.35 × 10^−9^; Figure S4F) in the Cavalli dataset.^37^

Most of these genetic alterations are in G4 samples, and a minority affect SHH tumors. Our Hi-C data gave us an opportunity to investigate potential mechanisms of inactivation of this gene. The Hi-C contact map of one of our G4 samples (MB3716) had a characteristic butterfly pattern that is consistent with a chromosomal inversion involving the region where *KDM6A* (MIM: 300128) is located (lower half of Figure 6D). As a comparison, we display the Hi-C contact map another G4 sample (MB3670) that does not appear to have this inversion (upper half of Figure 6B). The inversion was confirmed with linked-read (10× Genomics) whole-genome sequencing (Figure S4G; estimated breakpoints at chrX:26,926,652–26,928,652 and chrX:44,945,696–44,947,696). Using our RNA-seq data, we established that this inversion eliminates transcription of the 3′ exons of *KDM6A*. Transcription of all exons is clearly detected in non-inverted samples (Figure 6E). Our results are therefore consistent with an inversion resulting in truncation of *KDM6A*. This could be a previously unappreciated mechanism of *KDM6A* inactivation in G4 tumors and potentially other MB subtypes as well.

## Discussion

A wealth of Hi-C studies has profiled established cell lines to capture the main elements of their 3D genome organization. Some pioneering studies used this approach to define the main levels of genome organization in the nucleus.^16^ More recent studies have generated mechanistic insight into the functions of several key proteins and protein complexes that have major roles in 3D genome organization.^42^^,^^43^ Most of these studies have also taken advantage of established cell lines. There currently is a significant lack of understanding of genome structure in primary cancer samples, especially in pediatric brain tumors. Here, we have generated Hi-C data for 29 MB samples, representing 28 primary surgical specimens and one patient-derived primary cell culture. It is our hope that these datasets will be a resource to the community and will catalyze discoveries of epigenetic mechanisms that promote malignant transformation, tumor initiation, and progression.

Overall, our data largely support stability of genome organization between MB molecular subgroups at the global level. At the same time, however, our analyses also uncovered specific local differences in genome structure between different molecular subgroups of MB. For instance, we found that TAD boundary scores were the best Hi-C-derived metric to distinguish between G3, G4, and SHH samples. We present examples of TAD boundary shifts that are unlikely to be caused by SVs and that occur near genes that specify the cell lineage of origin of G3 and G4 tumors. These boundary shifts represented major restructuring of TAD structure and boundaries at the affected genomic regions. Except for these examples, the vast majority of TAD boundary changes did not correlate with the expression of known developmental genes or genes that have been clearly implicated with the etiology of these tumors. Globally, our results support the uncoupling of 3D genome structures—especially TADs—from gene transcription levels. The biological implications for the uncoupling of TAD structure and gene expression levels are puzzling but consistent with new literature.^44^^,^^45^

It is possible that epigenetic changes that affect chromatin accessibility on scales below the ones achieved with Hi-C are key to gene expression control. Alternatively, the underlying histone post-translational and DNA modifications might provide epigenetic memory independently of the underlying 3D genome organization.

It is also worth stressing that genome structure has been proposed to have roles other than regulating gene transcription. These alternative roles include other functional properties of the genome, including its replication timing and repair of mutations.^46^^,^^47^ Exploring the significance of TAD boundary shifts and differential strengths with respect to these alternative but major roles of the 3D genome across MB subgroups will be a focus of further studies.

The number of statistically significant differences in 3D genome features between subgroups may represent an underestimation. This is because we only studied clinical samples that are representative of differences between individuals. We see this as a strength of our study, but it needs to be acknowledged that the output of our analyses is necessarily more heterogeneous than if we worked with established cell lines. Furthermore, these samples are also likely to be characterized by intra-tumoral heterogeneity. Emerging approaches to perform single-cell Hi-C could help deconvolute 3D genome properties of distinct cell populations in MB subgroups.

The most dramatic changes in 3D genome structure were associated with SVs, as expected. Our analyses identified SVs in many samples, including at genes that have been previously shown to have important biological functions in MBs. It was previously hypothesized that SVs may have strong effects on gene transcription because of their ability to re-wire CREs and their target genes.^48^ Our direct measurements of the effects of SVs on 3D genome architecture (Hi-C data) and transcriptome (RNA-seq) indicate that most of the effects on transcriptional levels affected genes located at short distances from the SV breakpoints. Based on our data, it is important to emphasize that enhancer hijacking events are more likely to be the exception rather than the rule. Integration of epigenomic data (including ChIP-seq and ATAC-seq) with Hi-C and transcriptome data could therefore significantly expand the mechanistic insight of the role of epigenetic and genetic disruption on 3D genome architecture of cancer cells.

The Hi-C datasets we present here profile a collection of MB samples of clinical significance at high resolution. As the community is focused on better understanding disease mechanisms underlying MB subgroups, the focus is shifting from purely genetic studies to questions of how genetic and epigenetic forces co-operate to achieve cellular transformation and tumor development and progression. We expect that the Hi-C data described in this manuscript will nucleate further studies into genetic/epigenetic interactions, deepening our understanding of driver mechanisms. This is especially true for G3 and G4 MBs, for which our knowledge of crucial driver mechanisms is so far more limited.

## Data and code availability

Processed data including Hi-C contact matrices, RNA-seq counts, and derived data tables have been uploaded to the Gene Expression Omnibus (GEO) under series GSE246125. Raw sequencing data were uploaded to the European Genome-phenome Archive (EGA) under study ID EGAS50000000540. No new code was generated for this manuscript. Existing code and other computational tools used are associated with their respective citations and web links.

## Author contributions

J.J.Y.L., M.J.J., H.F., R.S., S.Y.T., H.C., M.Z., and N.J. contributed to the research activities described in this manuscript. W.A.W., N.J., J.R., M.D.T., and M.G. led and supervised the research described. J.J.Y.L., M.J.J., H.-M.C., M.D.T., and M.G. wrote the manuscript.

## Declaration of interests

The authors declare no competing interests.

## Supplemental information

Document S1. Figures S1–S4 and acknowledgments

Table S1. Excel file summarizing metadata

Table S2. Excel file containing quality control data for Hi-C contact map

Table S3. Excel file containing HiCCUPS output

Table S4. Excel file containing arrowhead output

Table S5. Excel file containing RobusTAD boundary calls

Table S6. Excel file containing differential boundaries between SHH and G3 subgroups

Table S7. Excel file containing differential boundaries between SHH and G4 subgroups

Table S8. Excel file containing differential boundaries between G4 and G3 subgroups

Table S9. Excel file containing differential list of differentially expressed genes near differential boundaries between SHH and G3

Table S10. Excel file containing set of G3 genes located near differential boundaries between G4 and G3

Table S11. Excel file containing set of G4 genes located near differential boundaries between G4 and G3

Table S12. Excel file listing recurrent structural variants inferred from Hi-C data Note: Unless otherwise stated, all genomic coordinates refer to human genome version hg38

Document S2. Article plus supplemental information

## References

[1] Northcott P.A., Korshunov A., Witt H., Hielscher T., Eberhart C.G., Mack S., Bouffet E., Clifford S.C., Hawkins C.E., French P. (2011). Medulloblastoma comprises four distinct molecular variants. J. Clin. Oncol. Off. J. Am. Soc. Clin. Oncol..

[2] Roussel M.F., Robinson G.W. (2013). Role of MYC in Medulloblastoma. Cold Spring Harb. Perspect. Med..

[3] Hendrikse L.D., Haldipur P., Saulnier O., Millman J., Sjoboen A.H., Erickson A.W., Ong W., Gordon V., Coudière-Morrison L., Mercier A.L. (2022). Failure of human rhombic lip differentiation underlies medulloblastoma formation. Nature.

[4] Kool M., Korshunov A., Remke M., Jones D.T.W., Schlanstein M., Northcott P.A., Cho Y.-J., Koster J., Schouten-van Meeteren A., van Vuurden D. (2012). Molecular subgroups of medulloblastoma: an international meta-analysis of transcriptome, genetic aberrations, and clinical data of WNT, SHH, Group 3, and Group 4 medulloblastomas. Acta Neuropathol..

[5] Northcott P.A., Buchhalter I., Morrissy A.S., Hovestadt V., Weischenfeldt J., Ehrenberger T., Gröbner S., Segura-Wang M., Zichner T., Rudneva V.A. (2017). The whole-genome landscape of medulloblastoma subtypes. Nature.

[6] Williamson D., Schwalbe E.C., Hicks D., Aldinger K.A., Lindsey J.C., Crosier S., Richardson S., Goddard J., Hill R.M., Castle J. (2022). Medulloblastoma group 3 and 4 tumors comprise a clinically and biologically significant expression continuum reflecting human cerebellar development. Cell Rep..

[7] Schwalbe E.C., Williamson D., Lindsey J.C., Hamilton D., Ryan S.L., Megahed H., Garami M., Hauser P., Dembowska-Baginska B., Perek D. (2013). DNA methylation profiling of medulloblastoma allows robust subclassification and improved outcome prediction using formalin-fixed biopsies. Acta Neuropathol..

[8] Ramaswamy V., Remke M., Bouffet E., Bailey S., Clifford S.C., Doz F., Kool M., Dufour C., Vassal G., Milde T. (2016). Risk stratification of childhood medulloblastoma in the molecular era: the current consensus. Acta Neuropathol..

[9] Shih D.J.H., Northcott P.A., Remke M., Korshunov A., Ramaswamy V., Kool M., Luu B., Yao Y., Wang X., Dubuc A.M. (2014). Cytogenetic prognostication within medulloblastoma subgroups. J. Clin. Oncol..

[10] Schwalbe E.C., Lindsey J.C., Nakjang S., Crosier S., Smith A.J., Hicks D., Rafiee G., Hill R.M., Iliasova A., Stone T. (2017). Novel molecular subgroups for clinical classification and outcome prediction in childhood medulloblastoma: a cohort study. Lancet Oncol..

[11] Louis D.N., Perry A., Wesseling P., Brat D.J., Cree I.A., Figarella-Branger D., Hawkins C., Ng H.K., Pfister S.M., Reifenberger G. (2021). The 2021 WHO Classification of Tumors of the Central Nervous System: a summary. Neuro Oncol..

[12] Vladoiu M.C., El-Hamamy I., Donovan L.K., Farooq H., Holgado B.L., Sundaravadanam Y., Ramaswamy V., Hendrikse L.D., Kumar S., Mack S.C. (2019). Childhood cerebellar tumours mirror conserved fetal transcriptional programs. Nature.

[13] Wechsler-Reya R.J., Scott M.P. (1999). Control of neuronal precursor proliferation in the cerebellum by Sonic Hedgehog. Neuron.

[14] Smith K.S., Bihannic L., Gudenas B.L., Haldipur P., Tao R., Gao Q., Li Y., Aldinger K.A., Iskusnykh I.Y., Chizhikov V.V. (2022). Unified rhombic lip origins of group 3 and group 4 medulloblastoma. Nature.

[15] Lieberman-Aiden E., van Berkum N.L., Williams L., Imakaev M., Ragoczy T., Telling A., Amit I., Lajoie B.R., Sabo P.J., Dorschner M.O. (2009). Comprehensive mapping of long-range interactions reveals folding principles of the human genome. Science.

[16] Rao S.S.P., Huntley M.H., Durand N.C., Stamenova E.K., Bochkov I.D., Robinson J.T., Sanborn A.L., Machol I., Omer A.D., Lander E.S., Aiden E.L. (2014). A 3D map of the human genome at kilobase resolution reveals principles of chromatin looping. Cell.

[17] Fudenberg G., Imakaev M., Lu C., Goloborodko A., Abdennur N., Mirny L.A. (2016). Formation of Chromosomal Domains by Loop Extrusion. Cell Rep..

[18] Sanborn A.L., Rao S.S.P., Huang S.-C., Durand N.C., Huntley M.H., Jewett A.I., Bochkov I.D., Chinnappan D., Cutkosky A., Li J. (2015). Chromatin extrusion explains key features of loop and domain formation in wild-type and engineered genomes. Proc. Natl. Acad. Sci. USA.

[19] Johnston M.J., Lee J.J., Hu B., Nikolic A., Hasheminasabgorji E., Baguette A., Paik S., Chen H., Kumar S., Chen C.C. (2024). TULIPs decorate the three-dimensional genome of PFA ependymoma. Cell.

[20] Durand N.C., Robinson J.T., Shamim M.S., Machol I., Mesirov J.P., Lander E.S., Aiden E.L. (2016). Juicebox Provides a Visualization System for Hi-C Contact Maps with Unlimited Zoom. Cell Syst..

[21] Durand N.C., Shamim M.S., Machol I., Rao S.S.P., Huntley M.H., Lander E.S., Aiden E.L. (2016). Juicer Provides a One-Click System for Analyzing Loop-Resolution Hi-C Experiments. Cell Syst..

[22] Li H., Durbin R. (2009). Fast and accurate short read alignment with Burrows-Wheeler transform. Bioinforma. Oxf. Engl..

[23] Wang Q., Li M., Wu T., Zhan L., Li L., Chen M., Xie W., Xie Z., Hu E., Xu S., Yu G. (2022). Exploring Epigenomic Datasets by ChIPseeker. Curr. Protoc..

[24] Yu G., Wang L.-G., He Q.-Y. (2015). ChIPseeker: an R/Bioconductor package for ChIP peak annotation, comparison and visualization. Bioinforma. Oxf. Engl..

[25] Wang B., Mezlini A.M., Demir F., Fiume M., Tu Z., Brudno M., Haibe-Kains B., Goldenberg A. (2014). Similarity network fusion for aggregating data types on a genomic scale. Nat. Methods.

[26] Kim D., Paggi J.M., Park C., Bennett C., Salzberg S.L. (2019). Graph-based genome alignment and genotyping with HISAT2 and HISAT-genotype. Nat. Biotechnol..

[27] Li H., Handsaker B., Wysoker A., Fennell T., Ruan J., Homer N., Marth G., Abecasis G., Durbin R., 1000 Genome Project Data Processing Subgroup (2009). The Sequence Alignment/Map format and SAMtools. Bioinforma. Oxf. Engl..

[28] Anders S., Pyl P.T., Huber W. (2015). HTSeq--a Python framework to work with high-throughput sequencing data. Bioinforma. Oxf. Engl..

[29] Ritchie M.E., Phipson B., Wu D., Hu Y., Law C.W., Shi W., Smyth G.K. (2015). limma powers differential expression analyses for RNA-sequencing and microarray studies. Nucleic Acids Res..

[30] Quinlan A.R., Hall I.M. (2010). BEDTools: a flexible suite of utilities for comparing genomic features. Bioinforma. Oxf. Engl..

[31] Riemondy K.A., Venkataraman S., Willard N., Nellan A., Sanford B., Griesinger A.M., Amani V., Mitra S., Hankinson T.C., Handler M.H. (2022). Neoplastic and immune single-cell transcriptomics define subgroup-specific intra-tumoral heterogeneity of childhood medulloblastoma. Neuro Oncol..

[32] Speir M.L., Bhaduri A., Markov N.S., Moreno P., Nowakowski T.J., Papatheodorou I., Pollen A.A., Raney B.J., Seninge L., Kent W.J., Haeussler M. (2021). UCSC Cell Browser: visualize your single-cell data. Bioinforma. Oxf. Engl..

[33] Ramírez F., Bhardwaj V., Arrigoni L., Lam K.C., Grüning B.A., Villaveces J., Habermann B., Akhtar A., Manke T. (2018). High-resolution TADs reveal DNA sequences underlying genome organization in flies. Nat. Commun..

[34] Lopez-Delisle L., Rabbani L., Wolff J., Bhardwaj V., Backofen R., Grüning B., Ramírez F., Manke T. (2021). pyGenomeTracks: reproducible plots for multivariate genomic datasets. Bioinforma. Oxf. Engl..

[35] Robinson J.T., Thorvaldsdóttir H., Winckler W., Guttman M., Lander E.S., Getz G., Mesirov J.P. (2011). Integrative genomics viewer. Nat. Biotechnol..

[36] Northcott P.A., Shih D.J.H., Peacock J., Garzia L., Morrissy A.S., Zichner T., Stütz A.M., Korshunov A., Reimand J., Schumacher S.E. (2012). Subgroup-specific structural variation across 1,000 medulloblastoma genomes. Nature.

[37] Cavalli F.M.G., Remke M., Rampasek L., Peacock J., Shih D.J.H., Luu B., Garzia L., Torchia J., Nor C., Morrissy A.S. (2017). Intertumoral Heterogeneity within Medulloblastoma Subgroups. Cancer Cell.

[38] Zwaig M., Johnston M.J., Lee J.J.Y., Farooq H., Gallo M., Jabado N., Taylor M.D., Ragoussis J. (2023). Linked-read based analysis of the medulloblastoma genome. Front. Oncol..

[39] Dali R., Bourque G., Blanchette M. (2018). RobusTAD: A Tool for Robust Annotation of Topologically Associating Domain Boundaries. bioRxiv.

[40] Dixon J.R., Selvaraj S., Yue F., Kim A., Li Y., Shen Y., Hu M., Liu J.S., Ren B. (2012). Topological domains in mammalian genomes identified by analysis of chromatin interactions. Nature.

[41] Robinson G., Parker M., Kranenburg T.A., Lu C., Chen X., Ding L., Phoenix T.N., Hedlund E., Wei L., Zhu X. (2012). Novel mutations target distinct subgroups of medulloblastoma. Nature.

[42] de Wit E., Nora E.P. (2023). New insights into genome folding by loop extrusion from inducible degron technologies. Nat. Rev. Genet..

[43] Nora E.P., Lajoie B.R., Schulz E.G., Giorgetti L., Okamoto I., Servant N., Piolot T., van Berkum N.L., Meisig J., Sedat J. (2012). Spatial partitioning of the regulatory landscape of the X-inactivation centre. Nature.

[44] Nora E.P., Goloborodko A., Valton A.-L., Gibcus J.H., Uebersohn A., Abdennur N., Dekker J., Mirny L.A., Bruneau B.G. (2017). Targeted Degradation of CTCF Decouples Local Insulation of Chromosome Domains from Genomic Compartmentalization. Cell.

[45] Rao S.S.P., Huang S.-C., Glenn St Hilaire B., Engreitz J.M., Perez E.M., Kieffer-Kwon K.-R., Sanborn A.L., Johnstone S.E., Bascom G.D., Bochkov I.D. (2017). Cohesin Loss Eliminates All Loop Domains. Cell.

[46] Akdemir K.C., Le V.T., Kim J.M., Killcoyne S., King D.A., Lin Y.-P., Tian Y., Inoue A., Amin S.B., Robinson F.S. (2020). Somatic mutation distributions in cancer genomes vary with three-dimensional chromatin structure. Nat. Genet..

[47] Marchal C., Sima J., Gilbert D.M. (2019). Control of DNA replication timing in the 3D genome. Nat. Rev. Mol. Cell Biol..

[48] Northcott P.A., Lee C., Zichner T., Stütz A.M., Erkek S., Kawauchi D., Shih D.J.H., Hovestadt V., Zapatka M., Sturm D. (2014). Enhancer hijacking activates GFI1 family oncogenes in medulloblastoma. Nature.

