# Supplementary material for: 3D genome topology distinguishes molecular subgroups of medulloblastoma
Source: Am J Hum Genet. 2024 Oct 30;111(12):2720–34. doi: 10.1016/j.ajhg.2024.10.003 (PMC11639096; doi:10.1016/j.ajhg.2024.10.003)
Supplement: Document S1. Figures S1–S4 and acknowledgments [file mmc1.pdf]

The American Journal of Human Genetics, Volume *111*

Supplemental information

3D genome topology distinguishes molecular subgroups of medulloblastoma

John J.Y. Lee, Michael J. Johnston, Hamza Farooq, Huey-Miin Chen, Subhi Talal Younes, Raul Suarez, Melissa Zwaig, Nikoleta Juretic, William A. Weiss, Jiannis Ragoussis, Nada Jabado, Michael D. Taylor, and Marco Gallo

Supplemental figures

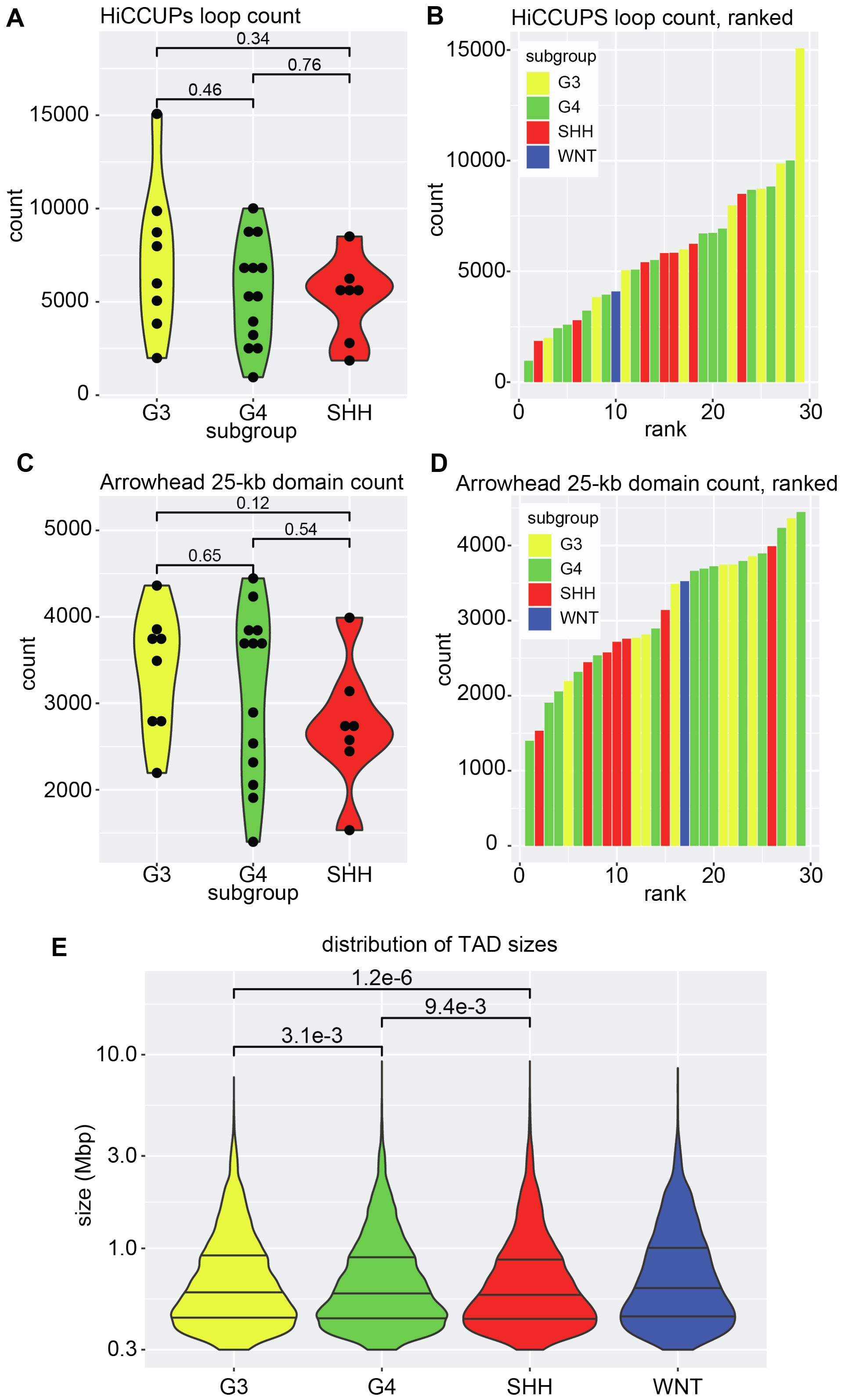

Figure S1. Quality metrics for Hi-C libraries.
(A) Count of loops in medulloblastoma subgroups. Each dot represents an individual sample.

(B) Ranking the loop counts in individual samples.
(C) Count of domains called with Arrowhead at 25 kb resolution. Each datapoint represents an individual sample.
(D) Ranking individual samples based on the counts of domains.
(E) TAD sizes in the four main medulloblastoma subgroups. Horizontal lines represent quartiles. P-values were computed with the Wilcoxon test.

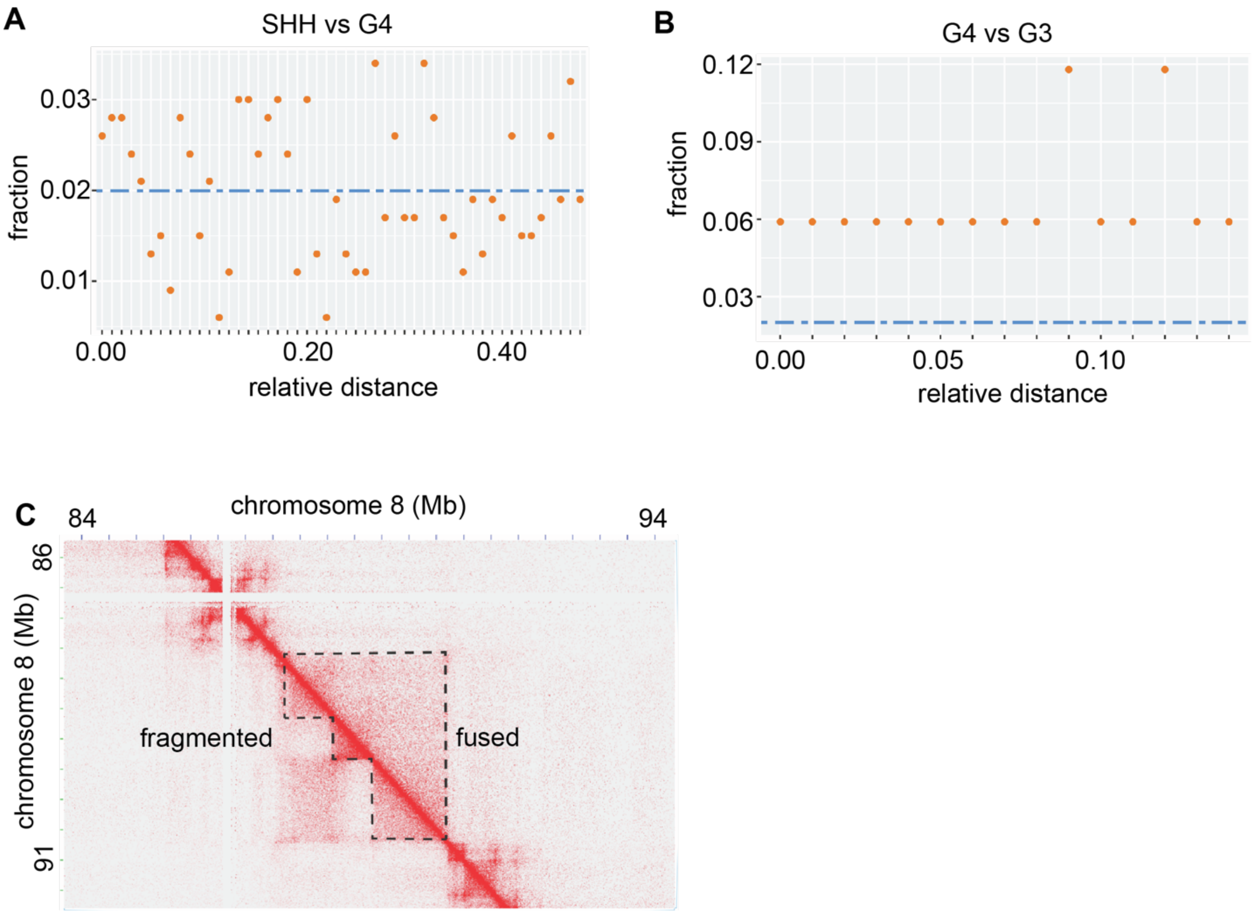

Figure S2. Differences in local 3D genome topology are not globally associated with differential gene expression.

(A) Relationship between differential gene expression and differential TAD boundaries between SHH and G3 medulloblastomas. Blue dashed line represents the expected frequency of observations based on 50 kb bins. Orange points represent the observed frequencies.

(B) Relationship between differential gene expression and differential TAD boundaries between G4 and G3 medulloblastomas.

(C) Example of a chromosomal region with largely different 3D genome structure in two samples.

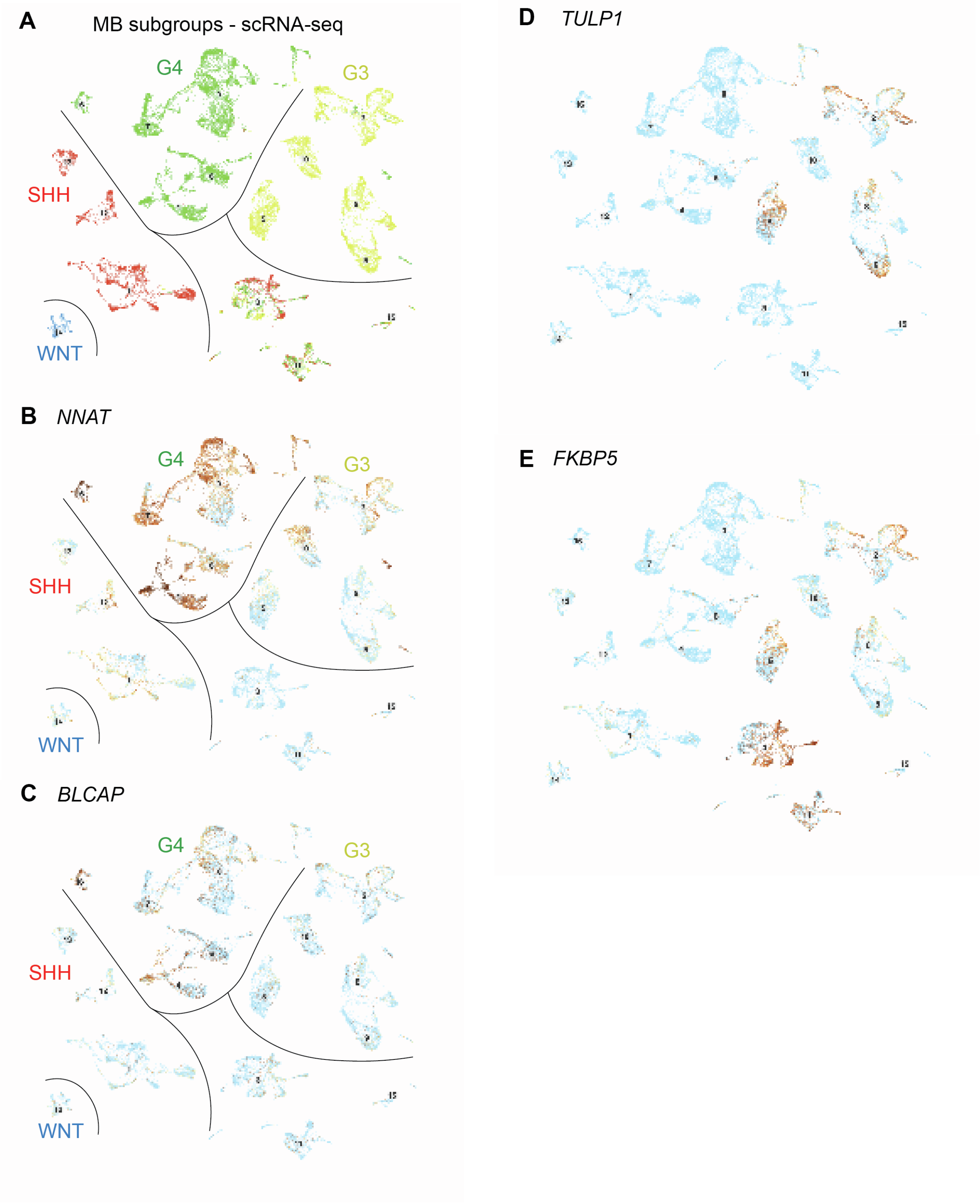
A
MB subgroups - scRNA-seq
G4
G3
SHH
WNT
B
NNAT
G4
G3
SHH
WNT
C
BLCAP
G4
G3
SHH
WNT
D
TULP1
E
FKBP5

Figure S3. Gene expression at the single-cell level.

(A) UMAP plot representing scRNA-seq data from a previously published dataset (GEO: GSE156053). The color of each cell was assigned according to its subgroup.

(B) Expression levels of *NNAT* in the scRNA-seq cohort. Shades of red correspond to higher expression levels, whereas light blue indicates no expression detected.

(C) *BLCAP* expression levels in the scRNA-seq cohort. Shades of red correspond to higher expression levels, whereas light blue indicates no expression detected.

(D) Expression levels of *TULP1* in a previously published scRNA-seq dataset (GEO: GSE156053).

(E) Expression levels of *FKBP5* in a previously published scRNA-seq dataset (GEO: GSE156053).

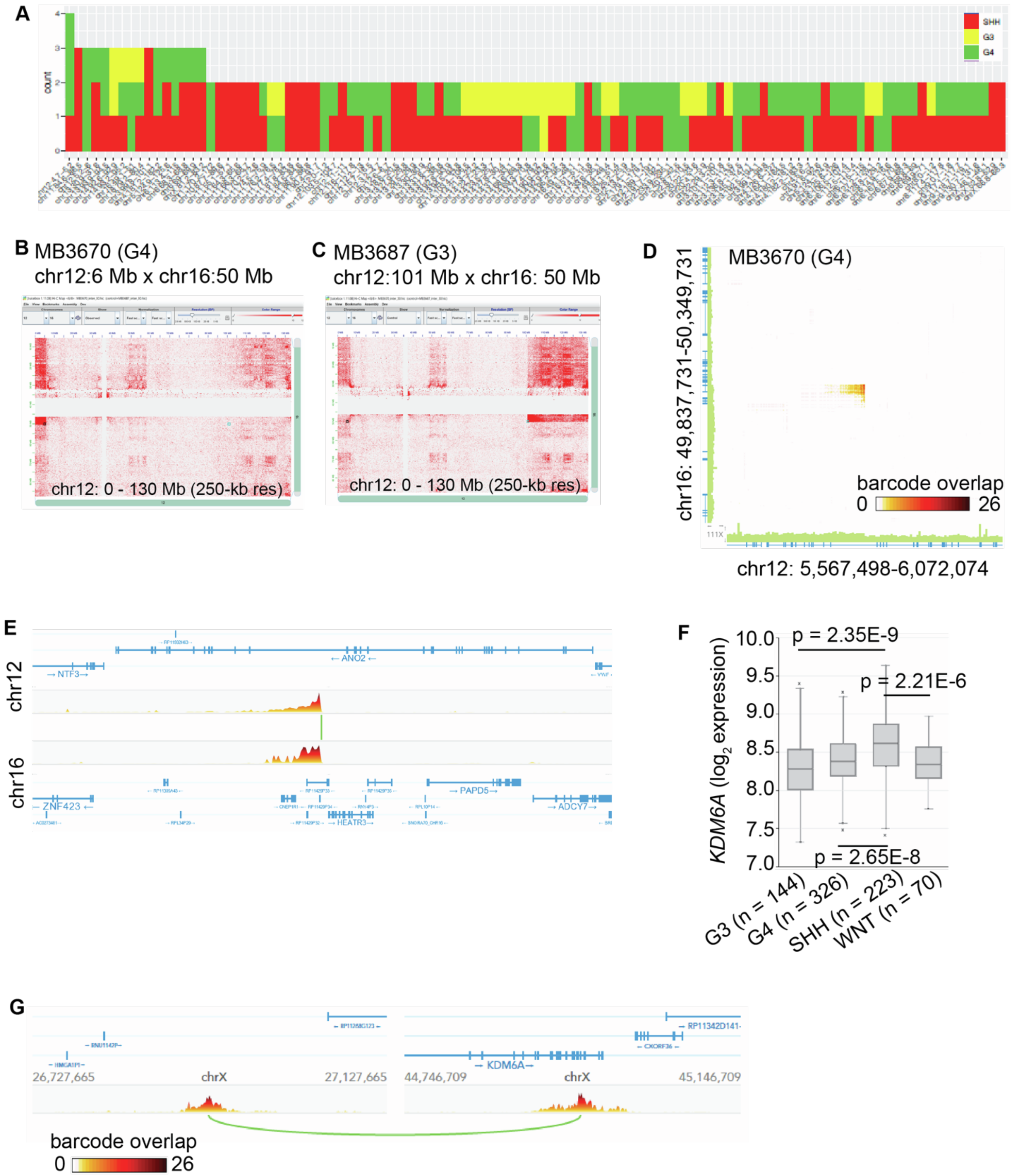

Figure S4.
(A) Chromosomal regions with putative structural variants based on inferences from the Hi-C data for individual medulloblastoma samples in our cohort.
(B) Hi-C contact map supporting a t(12; 16) translocation in MB3670.
(C) Hi-C contact map supporting a t(12; 16) translocation in MB3687.
(D) Matrix view of linked-read whole genome sequencing data for sample MB3670. Barcode overlap showcases a translocation that was inferred with Hi-C data.

(E) Linear view of linked-read whole genome sequencing data for MB3670. The barcode overlap supports the existence of a translocation between chromosomes 12 and 16. This is an alternative view of data presented in (D).

(F) Transcriptional levels of *KDM6A* in the four major medulloblastoma subgroups. Data from. The Cavalli et al datasets (PMID: 28609654) were re-analyzed with R2 to generate this plot. P-values were generated with pair-wide Welch t-test.

(G) Linked-read whole genome sequencing supports an inversion of chromosome X that results in disruption of the *KDM6A* locus.

Supplemental tables

Table S1. Excel file summarizing metadata.

Table S2. Excel file containing quality control data for Hi-C contact map.

Table S3. Excel file containing HiCCUPS output.

Table S4. Excel file containing arrowhead output.

Table S5. Excel file containing RobusTAD boundary calls.

Table S6. Excel file containing differential boundaries between SHH and G3 subgroups.

Table S7. Excel file containing differential boundaries between SHH and G4 subgroups.

Table S8. Excel file containing differential boundaries between G4 and G3 subgroups.

Table S9. Excel file containing differential list of differentially expressed genes near differential boundaries between SHH and G3.

Table S10. Excel file containing set of G3 genes located near differential boundaries between G4 and G3.

Table S11. Excel file containing set of G4 genes located near differential boundaries between G4 and G3.

Table S12. Excel file listing recurrent structural variants inferred from Hi-C data.

Note: Unless otherwise stated, all genomic coordinates refer to human genome version hg38.

ACKNOWLEDGMENTS

M.D.T. and W.A.W. are supported by the NIH (R01NS106155, R01CA159859 and R01CA255369). M.D.T. is a CPRIT Scholar in Cancer Research and is supported by The Pediatric Brain Tumour Foundation, The Terry Fox Research Institute, The Canadian Institutes of Health Research (CIHR), The Cure Search Foundation, Matthew Larson Foundation (IronMatt), b.r.a.i.n.child, Meagan's Walk, SWIFTY Foundation, The Brain Tumour Charity, Genome Canada, Genome BC, Genome Quebec, the Ontario Research Fund, Worldwide Cancer Research, V-Foundation for Cancer Research, and the Ontario Institute for Cancer Research through funding provided by the Government of Ontario. M.D.T. is also supported by a Canadian Cancer Society Research Institute Impact grant, a Cancer Research UK Brain Tumour Award, and by a Stand Up To Cancer (SU2C) St. Baldrick's Pediatric Dream Team Translational Research Grant (SU2C-AACR-DT1113) and SU2C Canada Cancer Stem Cell Dream Team Research Funding (SU2C-AACR-DT-19-15) provided by the Government of Canada through Genome Canada and CIHR, with supplementary support from the Ontario Institute for Cancer Research through funding provided by the Government of Ontario. Stand Up to Cancer is a program of the Entertainment Industry Foundation administered by the American Association for Cancer Research. M.D.T. is also supported by the Garron Family Chair in Childhood Cancer Research at the Hospital for Sick Children and the University of Toronto. J.J.Y.L is supported by University of Toronto Fellowship and Ontario Graduate Scholarship. This work was also supported by the Brain Canada Foundation through the Canada Brain Research Fund, with the financial support of Health Canada and the Azrieli Foundation through an Azrieli Future Leader in Canadian Brain Research grant to M.G.; CIHR project grants (PJT-156278 and PJT-173475) to M.G.; a Canada Research Chair to M.G. M.J.J was supported by a CIHR postdoctoral fellowship.
